# Postponement of the Third American Medical Congress

**Published:** 1898-12

**Authors:** Charles A. L. Reed

**Affiliations:** Secretary; Cincinnati


### Postponement of the Third American Medical Congress.

CINCINNATI, Nov. 5, 1898.

MY DEAR SIR:—I have the honor to annuounce that in April, 1898, I received from Dr. Jose Manuel de los Rios, Chairman of the Committe on Organization of the III Pan-American Medical Congress, a request that, in consequence of the then existing rebellion in Venezuela, no definite arrangements be made at that time relative to the meeting of the Congress previously appointed to be held in Caracas in December, 1899.

The following communication relative to the same subject is just at hand:

CARACAS, September 25, 1898.

*Dr. Charles A. L. Reed, Secretary of the International Executive Commission, Cincinnati, Ohio.*

DEAR SIR:—After having sent my communication dated April last, I find it to be my duty to notify you that, although the considerations pointed out in it have already ended, our country has been scourged by small pox which has taken up all our physicians' activities and time, depriving them of going into scientific works. And, as that state of mind of our people and government after such calamities as war and epidemic, would greatly interfere with the good success of our next meeting, I beg leave to tell you, in order you will convey it to the International Executive Committee, that our Government and Commission would be grateful to have the meeting which was to take place in Caracas in December, 1899, adjourned for one year later. I am, dear Doctor,

Yours respectfully,

THE PRESIDENT.

[Signed] DR. JOSE MANUEL DE LOS RIOS.

In accordance with the request of the Government of Venezuela, and of the Committee on Organization, the III Pan-American Medical Congress is hereby postponed to meet in Caracas in December, 1900.

For the International Executive Committee.

CHARLES A. L. REED, Secretary.

